# A road traversing a protected area has little effect on feeding and foraging behaviour of yellow baboons

**DOI:** 10.1002/ece3.9405

**Published:** 2022-12-04

**Authors:** Amani Kitegile, Shombe N. Hassan, Guy W. Norton

**Affiliations:** ^1^ Department of Wildlife Management Sokoine University of Agriculture Morogoro Tanzania; ^2^ Animal Behaviour Research Unit Morogoro Tanzania

**Keywords:** dietary composition, exotic food, feeding behavior, highway in protected areas, TANZAM highway, yellow baboons

## Abstract

The Tanzania–Zambia (TANZAM) Highway traversing Mikumi National Park (MINAPA) has been a concern for wildlife managers since it was first paved in 1973–1974. After its upgrade in 1989–1990, researchers have documented increasing traffic resulting in considerable animal injuries and mortalities. Yellow baboons (*Papio cynocephalus*) in MINAPA use the road as the bridge to and from foraging areas, therefore in addition to the risk of mortality road use could potentially have significant influence on their feeding behavior. However, knowledge on the influences of the TANZAM highway in the feeding behavior of yellow baboons is sparse. Using focal animal sampling techniques, we collected data on feeding and foraging behavior of two habituated troops of yellow baboons to examine to what extent the TANZAM highway is important in their feeding and foraging behavior. Results showed that in relation to habitat availability, visitation to habitat types reflect actual habitat choice of baboons. In general, yellow baboons less frequently visit and spent less time on the highway than natural habitats. Whenever they were on the highway, adult females and subadult males engage more into feeding, resting and socializing, while adult males were more vigilant. The major dietary compositions were fruits, seeds, leaves, sap, and invertebrates, almost exclusively collected from natural habitats, foods from the highway were opportunistically consumed. This study provides empirical evidence and concludes that yellow baboons do not directly depend on the highway for food, rather they use the TANZAM highway as normal part of their home range. However, its location near sleeping sites may have significant impact on baboons' activity budget. With these findings, we recommend strict implementation of rules against park littering and animal feeding in protected areas traversed by highways.

## Graphical abstract

Yellow baboons do not directly depend on the highway for food, rather they use it to improve visibility and reduces the perceived predation risk compared to natural habitats.

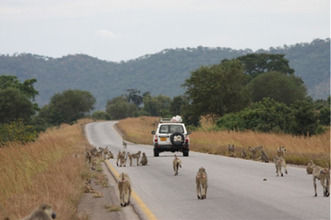

## 1 INTRODUCTION

Human advancement has led into rapid development of infrastructure, particularly road networks. As a result, large road networks drastically alter landscapes in which they pass through (Forman & Alexander, 1988; Garriga et al., 2012). The geographical location and the landscapes within and surrounding some protected areas results in national, provincial, and local roads being built through these protected areas; thus saving as transport corridors (Barrueto et al., 2014). Studying the ecological effect of roads within protected areas is important for improving species conservation because road effects often extend far beyond the surface covered by the road itself (Forman & Alexander, 1988; Monsuroglu et al., 2013). Roads that traverse protected areas can directly or indirectly affect wildlife populations in a number of ways, both during and after construction (Bennet et al., 2011; Forman & Alexander, 1988). During construction, roads directly affect wildlife populations by destroying habitats and fragmenting them, consequently restricting animal movements and access to critical resources (Forman & Alexander, 1988; Monsuroglu et al., 2013). After construction, roads can affect wildlife populations through road kill (Vos & Chardon, 1998) and may alter patterns of their natural behavior more especially feeding behavior and contact with human (Laurence et al., 2009; Orams, 2002).

Mikumi National Park (MINAPA) is the only protected area in Tanzania traversed by the A‐7 highway (Tanzania–Zambia Highway‐TANZAM) (Newmark et al., 1996). TANZAM highway was first paved in 1973–1974 and ultimately tarmacked in 1989–1990, and since then wildlife road mortality has been systematically recorded. For instance, from 1973–1988 road mortalities were recorded to be 456 large mammals (Newmark et al., 1996), and this figure increased to 2241 animals between 1992 and 2008 (Albert Mziray, *pers. comm*.). Currently, road mortalities have been estimated to be over 360 animals per annum (Julius Keyyu, *pers. comm*.), with yellow baboons (*Papio cynocephalus*) being one of the species killed by motor vehicles. Regular users of TANZAM highway associated yellow baboons' road mortality with their frequent occurrence along the highway in pursuit of food. Moreover, a news article by Robi (2015) speculated that yellow baboons intentionally visit the highway to access food thrown by passengers. However, there is no empirical evidence to support this speculation.

Our objective was, therefore, to provide first hand evidence on the potential of TANZAM highway on feeding and foraging behavior of yellow baboons in MINAPA. In that regard, we put forward three hypotheses related to the use of highway by yellow baboons in MINAPA; first we hypothesized that: (1) baboons used the highway for ease of movement through parts of their home range; second: (2) baboons used the highway because improved visibility reduces predation risk when moving within the home range and, third: (3) high quality, easy to process domestic food items made it energetically profitable to travel and feed along the highway.

## 2 MATERIALS AND METHODS

### 2.1 Description of the study area

This study was conducted in MINAPA within the central floodplain and the eastern hills, covering an area of about 135 km^2^. Data were collected from September 2010 to November 2012, covering two ecological years (two dry and two rainy seasons). The research work complied with the ethical protocol for conducting research on wild animals approved by Tanzania Wildlife Research Institute (TAWIRI). Moreover, the research adhered to the legal requirement of conducting research in national parks, as provided by Tanzania National Parks Authorities (TANAPA).

**FIGURE 1:**
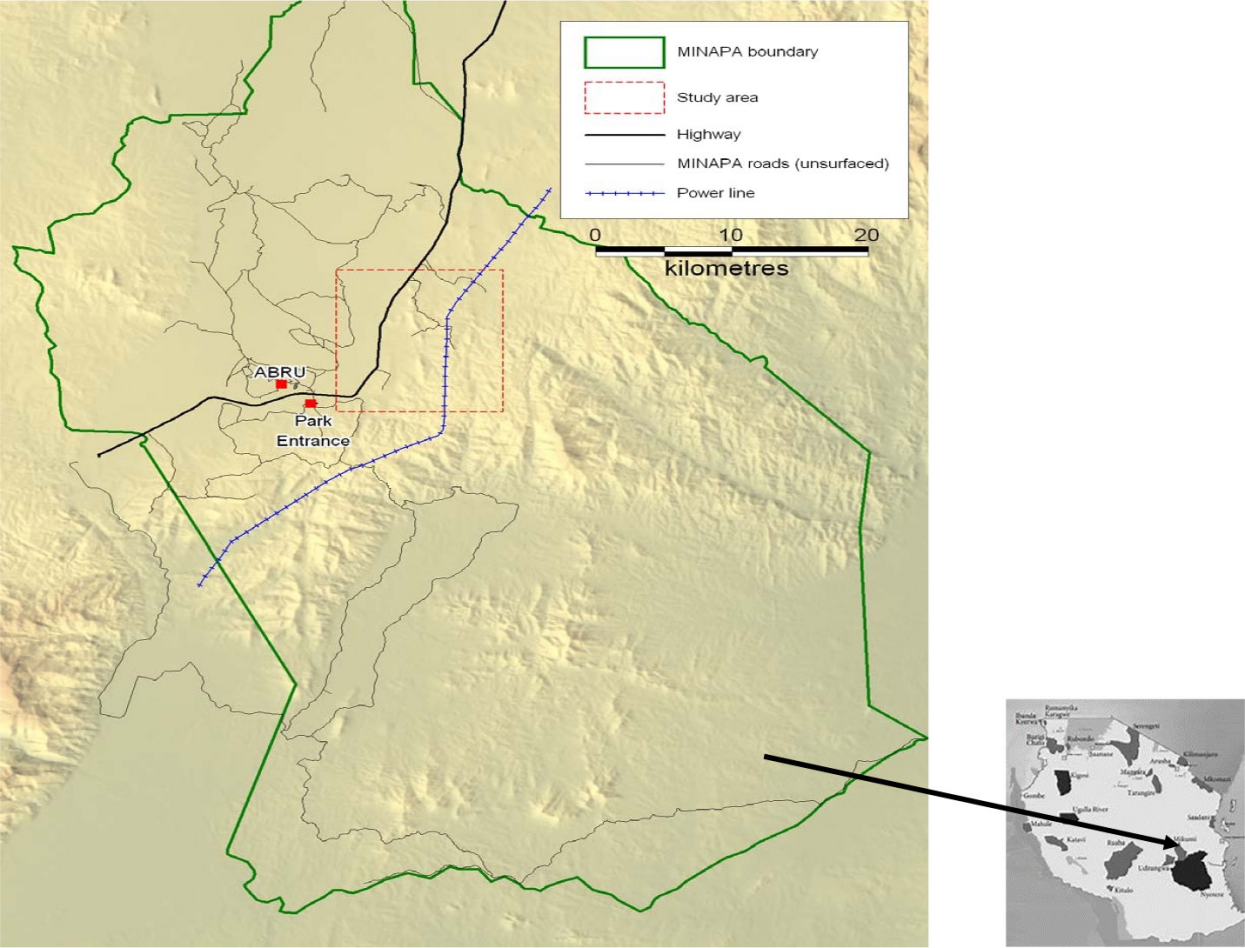
A map of Mikumi National Park (MINAPA) indicating the location of the Viramba troop range (black rectangle); an insert is a map of Tanzania indicating the location of Mikumi National Park within the country.

**FIGURE 2:**
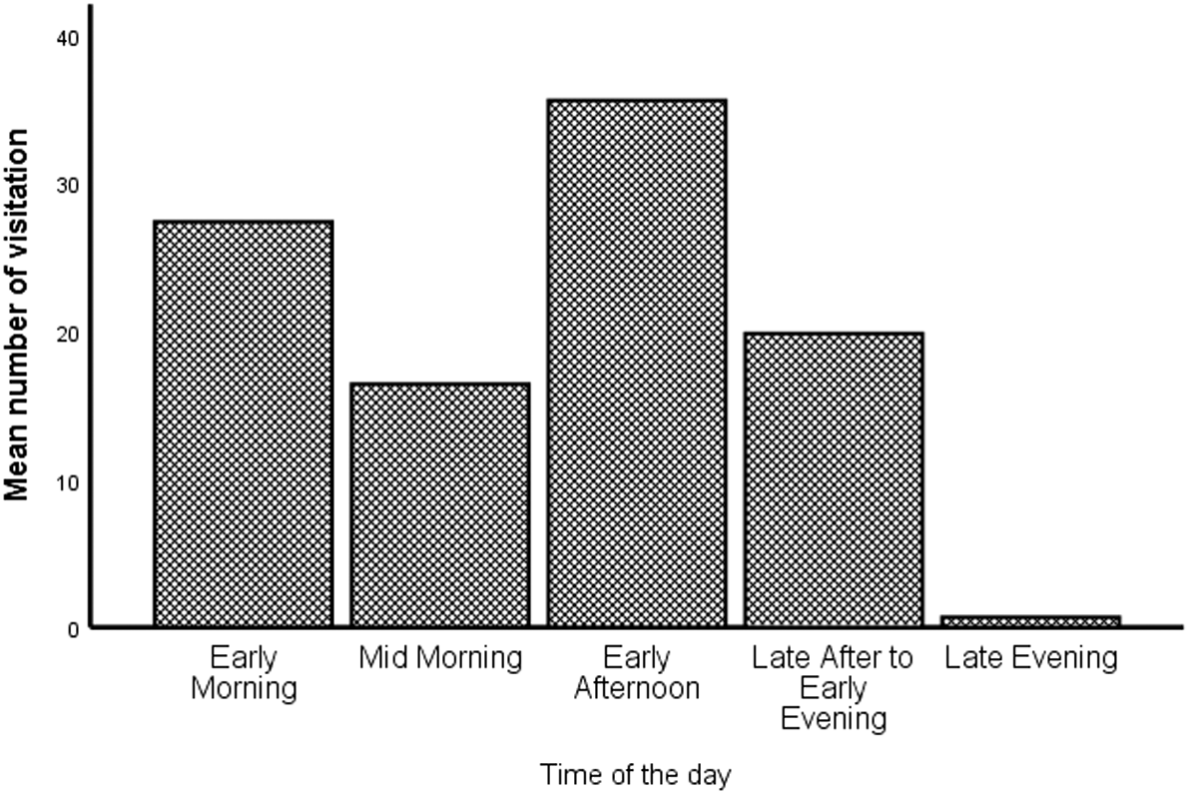
Mean number of yellow baboons' observations on the highway at different periods of the day.

The area where the research was conducted (Figure 2) serves as the core range for the habituated troops of yellow baboons, known as the Viramba troops living within MINAPA. The troops range extends from the Mgoda River to the park main gate in north–south direction, and 2 km East of the power line to Mkata River in an East–West direction (Norton et al., 1987). This area is traversed by the Tanzania–Zambia pipeline (TAZAMA pipeline), and the TANZAM highway, which runs the study area for about 10 km and passes close to most of the two troops sleeping sites (Hawkins, 1999; Norton et al., 1987) (Figure 1).

The vegetation types in the area are grassland, wooded grassland, and woodland (predominantly of open nature), which are mostly dominated by either *Brachystegia*, *Combretum* or *Terminalia* spp. Riverine forests occurring in narrow strips along river courses (Korongos) provide the baboons with potential tree refuges used as sleeping sites (Norton et al., 1987) (Figure 2).

### 2.2 Study subjects

Yellow baboons studied for this research belong to well‐habituated troops called Viramba. The original Viramba troop (V0) was habituated in 1975 and has been observed and studied ever since. For the past four decades, the Viramba study animals have undergone a number of fission and fusion events, accompanied with changes in population size (ABRU *unpublished records*). The first split was in 1978 when V0 split into two troops, V1 and V2. Troop fissions and/or fusions continued in 1979, 1989, 1994 and the last split was in August 2010 few months before commencement of this study, when the V4 split into V5 and V6 from which data for this study was collected (Kitegile, 2017).

### 2.3 Sampling and data collection

Focal animal sampling technique (animal follow) was used for data collection from study subjects, which were adult males, adult females, and subadult males from two Viramba troops; Viramba 5 (V5, 19 individuals in 2010; 15 individuals in 2012) and Viramba 6 (V6, 29 individuals in 2010; 25 individuals in 2012). The selection of study subjects was done to examine influence of sex and/or body size dimorphism on the foraging and feeding behavior of baboons (Kitegile, 2017). Follows were conducted daily from about 7:00 a.m. when troops descend from sleeping sites to 5:00 p.m. when they retire to sleeping sites. A day was divided into four periods of 2.5 h each. In each time period, a single subject was followed for 16 min, which was divided into eight intervals of 2 min each. Individuals in a single troop were followed for four continuous days in a week then observations were switched to the other troop. Focal animals were randomly selected by picking up a single name from a container. The chosen name was listed for focal animal follows in one of the four periods of the day. A maximum of six subjects were followed in each given period of a day (Kitegile, 2017).

Feeding data including plant and animal species consumed, parts consumed, and package (size of food part consumed) were recorded during focal animal sampling using the one–zero technique (Suen and Ary, 1984). Other information such as age and sex class of focal subjects, date of data collection (in Julian calendar), seasonal quarter, time of day, location (GPS), habitat type, and weather condition were recorded at the onset of focal animal follows. Habitat types were categorized as natural habitats (woodland, grassland, wooded grassland, and riverine forests) and highway.

### 2.4 Data analyses

Focal animal follow was used as the basic unit of analysis; therefore, baboons' visitation in different habitat types were analyzed as total number of follows baboons were recorded in a particular habitat type. The frequency of feeding in a given habitat was analyzed as the proportion with which feeding was recorded within focal samples relative to all other behaviors in that habitat. Amount of feeding in a given habitat was analyzed as relative percentage of all feeding follows in all habitats.

Time spent in different habitats was analyzed by converting the number of follows in that particular habitat into minutes of follows, and multiplying the resulting minutes values of follows by 16 (which was the duration in minutes for a single follow), and then converting the minutes of follows into hours by dividing them by 60.

The proportions of time within a single follow (i.e., percent of intervals in 16 min) baboon spent on consuming a food part was considered to reflect contribution of that food item in the overall diet. Therefore, we calculated the percent of two‐minute intervals a subject spent feeding on a particular food per feeding follow. The value was calculated using the following formula:

$$P=\left(\left[\sum_{1=8}/N\right]\times 100\right).$$

Where: *P* = Percentage contribution of food part; ∑_1=8_ = Sum of feeding intervals a particular food part was consumed in a single feeding follow; *N* = total number of feeding intervals.

Wald Chi‐Square statistics was performed in the Generalized Estimate Equation (GEE) for the significance impact of response variables (age‐sex classes, occurrence of vigilance) on predictor variables. Response variable was assumed to be a binomial distribution with trial size of 8, and Binary logistic was used as model logit link function.

## 3 RESULTS

### 3.1 Baboon visitation in different habitats

For 270 days from 2010 to 2012, we followed yellow baboons in a total of 3838 counts and collected data from 68 study subjects comprised of 33 adult females, 22 adult males, and 13 subadult males in two different troops; followed adult females were of both lower and higher‐ranking subjects. On average, each study subject was followed in 54 follows, ranging between 2 and 116 follows per study animal. No subjects were lost to road mortality during the study period.

Yellow baboons were recorded visiting five different natural habitat types (Table 1) and the highway within their home range. More than half (52%) of yellow baboons' visitation to different habitats were in wooded grassland, and 3.9% (148 follows) of visitation records were on the highway (Table 1).

**TABLE 1:** Yellow baboons' visitation to different habitats presented in number of follows and proportion of follow (in percentages).

Habitat	Estimated Area (km^2^)	Proportion of Covered Area (%)	Number of follows	Proportion of follow (%)
Grassland	58.9	43.6	242	6.3
Wooded grassland	18.9	14.0	1997	52.0
Open woodland	12.2	9.0	695	18.1
Woodland	40.5	30.0	236	6.1
Riverine forest	4.0	3.0	520	13.6
Total Natural Habitats	134.5	99.6	3690	96.1
Highway	0.5	0.4	148	3.9
Overall Total	135.0	100.0	3838	100.0

*Note*: The estimated size of each habitat and its proportion coverage (in percentage) of the overall study site (Viramba range = 135 km^2^).

The frequency of follow observation on the highway (visitation) varied between periods of the day. Yellow baboons' visitation to the highway were most frequently observed from early in the morning (07h00–09h00) and early afternoon (12h00–14h29). Fewer visitations were observed in mid‐morning (09h30–11h59) and late afternoon (14h30–17h00). Highway visitations were less observed in the short late evening period (17h30–18h00) (Figure 2).

**FIGURE 3:**
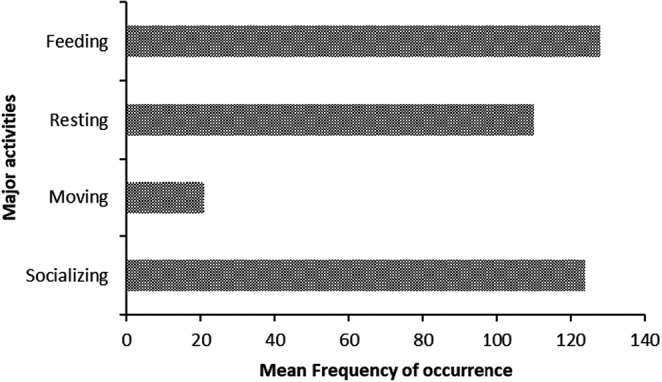
Frequency of follows on the highway of four major activities performed by yellow baboons

**FIGURE 4:**
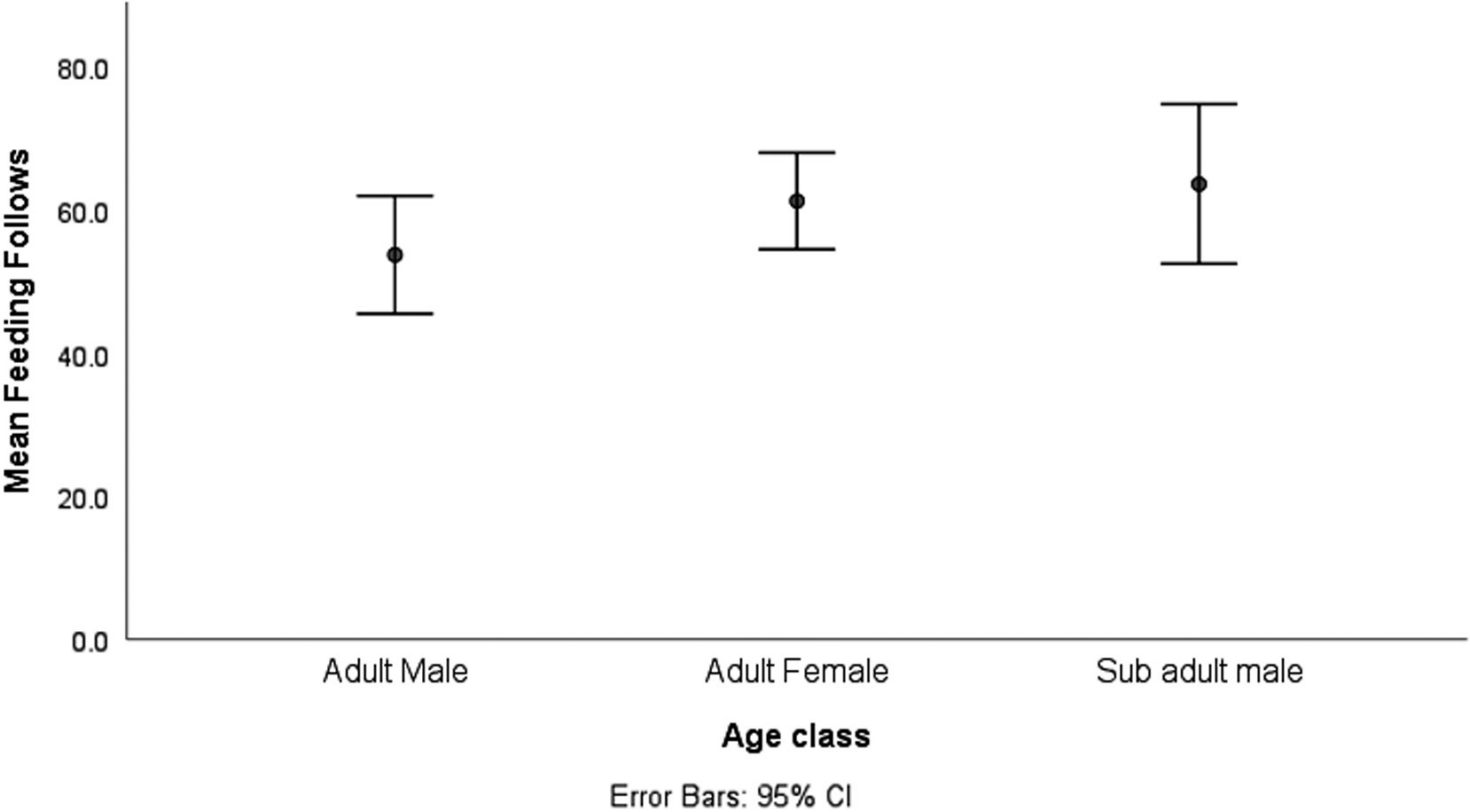
Percent of total highway follows where feeding occurred for age‐sex classes

When on the highway yellow baboons engaged into different activities, and the most frequently performed activities were feeding, resting, moving, and socializing (Figure 3). While age‐sex classes were found to have no significant influence on activities performed by baboons when they were on the highway (GEE Wald $X_{2}^{2}$ = 3275.2, *N* = 148, *p* > .05), subadult males and adult females were recorded feeding most frequently than adult males on the highway (Figure 4).

### 3.2 Time spent in different habitat types

In general, yellow baboons spent more time in natural habitats than on the highway; out of approximately 1023 observational hours; baboons spent a total of 984 h (96.2% of observation time) in natural habitats, and 38.9 h (3.8% of observation time) on the highway (Table 2). Within habitats, baboons spent more than half of the time in wooded grassland, which covers only 14% of their total home range, and 3.8% of the time on highway, which covers less than 0.5% of home range, while they spent a total of 12.4% of the time in grassland and woodland, which together cover about 74% of their home range (Table 2). Moreover, despite varied time spent in different vegetation in natural habitat, and on the highway, baboons spent relatively similar proportion of time feeding in these habitats except for riverine forest (Table 2).

**TABLE 2:** Total time spent and time spent feeding (in hours) by yellow baboons in different habitats, and proportion (in percentages) of time spent feeding in each habitat, with estimated size and proportion coverage of each habitat in baboons’ home range.

Habitat	Estimated area of home range covered (km^2^)	Proportion of covered area (%)	Observation time (h)		Proportion of time spent feeding (%)	Proportion of time spent in habitats (%)
			Total time	Feeding time		
Grassland	58.9	43.6	64.5	58.1	90.1	6.3
Wooded grassland	18.9	14.0	532.5	439.5	82.5	52.1
Open woodland	12.2	9.0	185.3	150.7	81.3	18.1
Woodland	40.5	30.0	62.9	53.6	82.5	6.1
Riverine	4.1	3.0	138.7	82.7	59.6	13.6
Total Natural habitats	134.5	99.6	984.0	784.5		96.2
Highway	0.5	0.4	38.9	34.7	89.3	3.8
Total all habitats	135	100	1022.9	819.2		100

**FIGURE 5:**
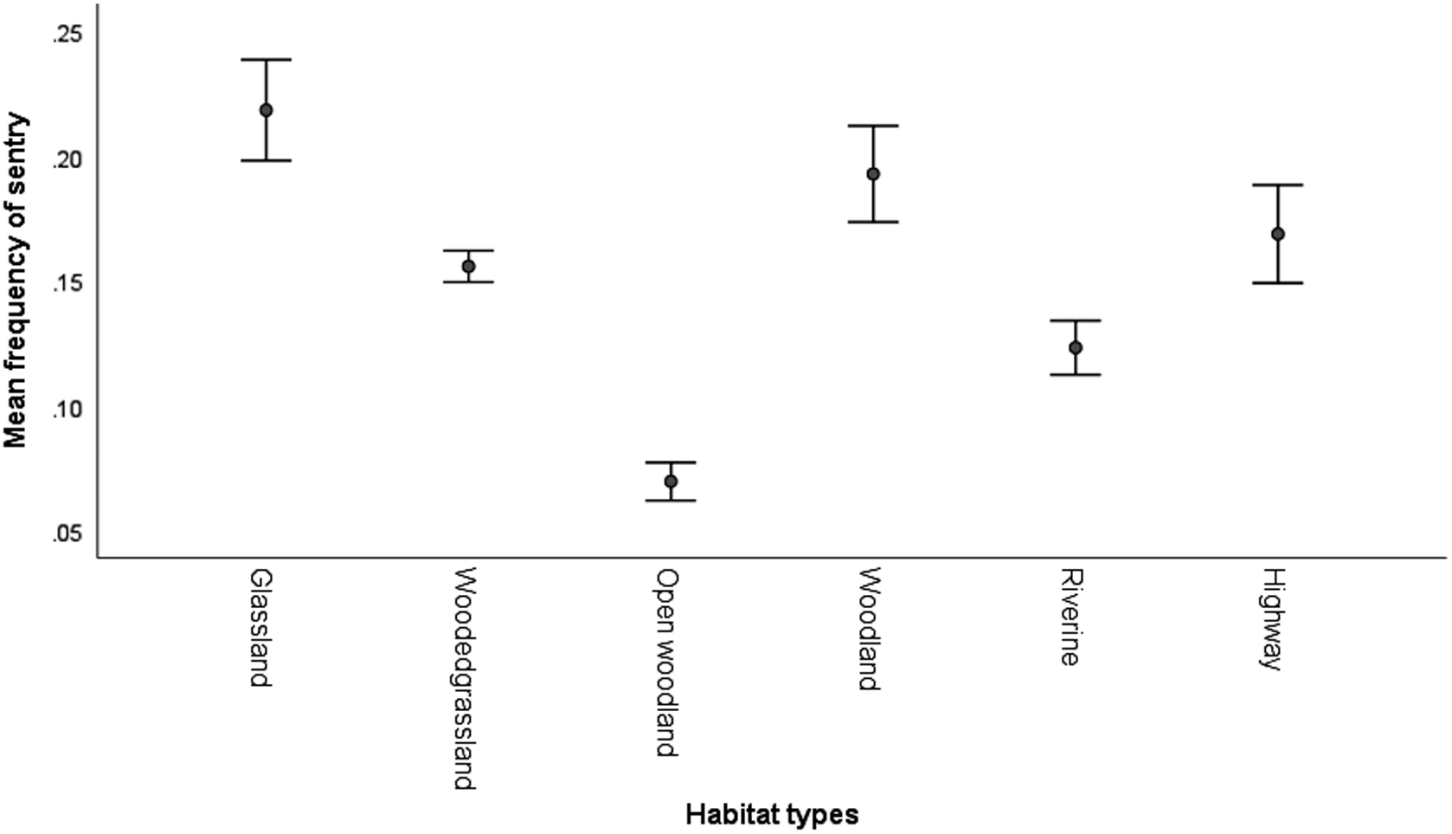
Mean frequency of vigilance (sentry) by yellow baboons when feeding in different habitats

**FIGURE 6:**
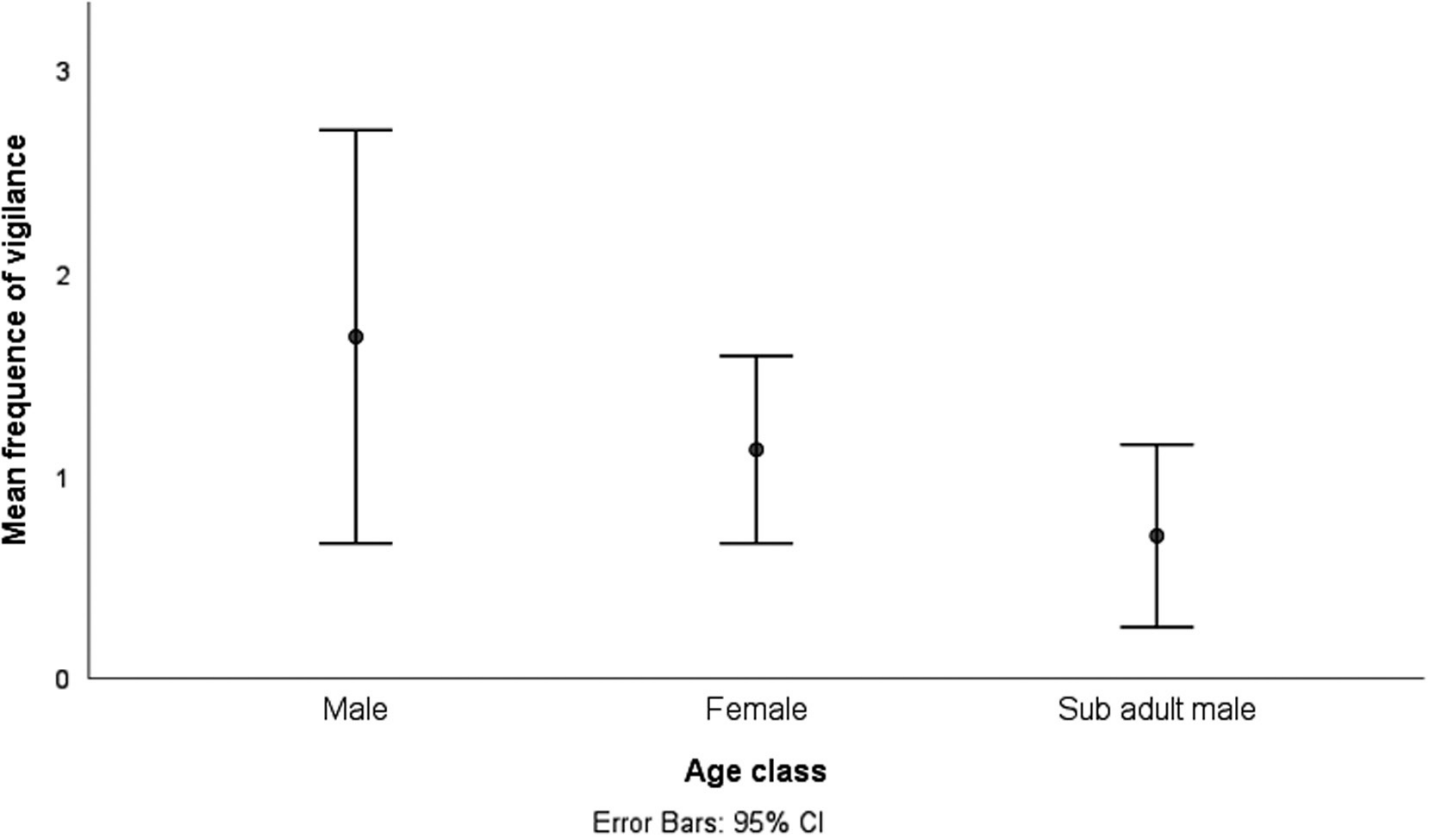
Mean frequency of yellow baboons; vigilance by age‐sex classes when feeding on the highway

Further analysis was conducted on vigilance behavior of baboons when feeding on the highway and in natural habitats. The frequency of vigilance during feeding significantly differ between habitats (GEE Wald $X_{5}^{2}$ = 1450.5, *N* = 30704, *p* < .05). Yellow baboons were more vigilant when feeding in grassland and woodland, and had similar levels of vigilance on wooded grassland and on the highway. Baboons were least vigilant when feeding in riverine forests and open woodland (Figure 5). Of the three age‐sex classes, adult males were recorded to be more vigilant when feeding on the highway than adult females and subadult males (Figure 6). However, vigilance behavior when feeding on the highway was not influenced by age‐sex classes (GEE Wald $X_{2}^{2}$ = 198.4, *N* = 148, *p* > .05).

### 3.3 Food composition

Yellow baboons were recorded feeding on a wide variety of food including natural foods such as wild plants, fungus, vertebrates, and invertebrates, and human‐derived food/exotic food such as agricultural produce spill and/or left overs thrown on the highway from vehicles. In general, baboons' diet of both age‐sex classes was mainly composed of plant food than animal and human‐derived/exotic foods (Table 3).

**TABLE 3:** Overall number of feedings follows and feeding follows per food source with proportion of food sources used (as percentages in blanket) by age‐sex category.

Age‐sex classes	Overall Feeding follows	Food sources specific feeding follows (feeding proportion %)		
		Plants	Animals	Exotics foods
Adult Male	557	507 (91%)	180 (32.3%)	29 (5.2%)
Adult Female	1862	1793 (96.3%)	558 (30%)	69 (3.7%)
Subadult Male	655	635 (97%)	175 (26.7%)	24 (3.7%)
Total	3074	2935 (95.6%)	913 (29.7%)	122 (3.9%)

**FIGURE 7:**
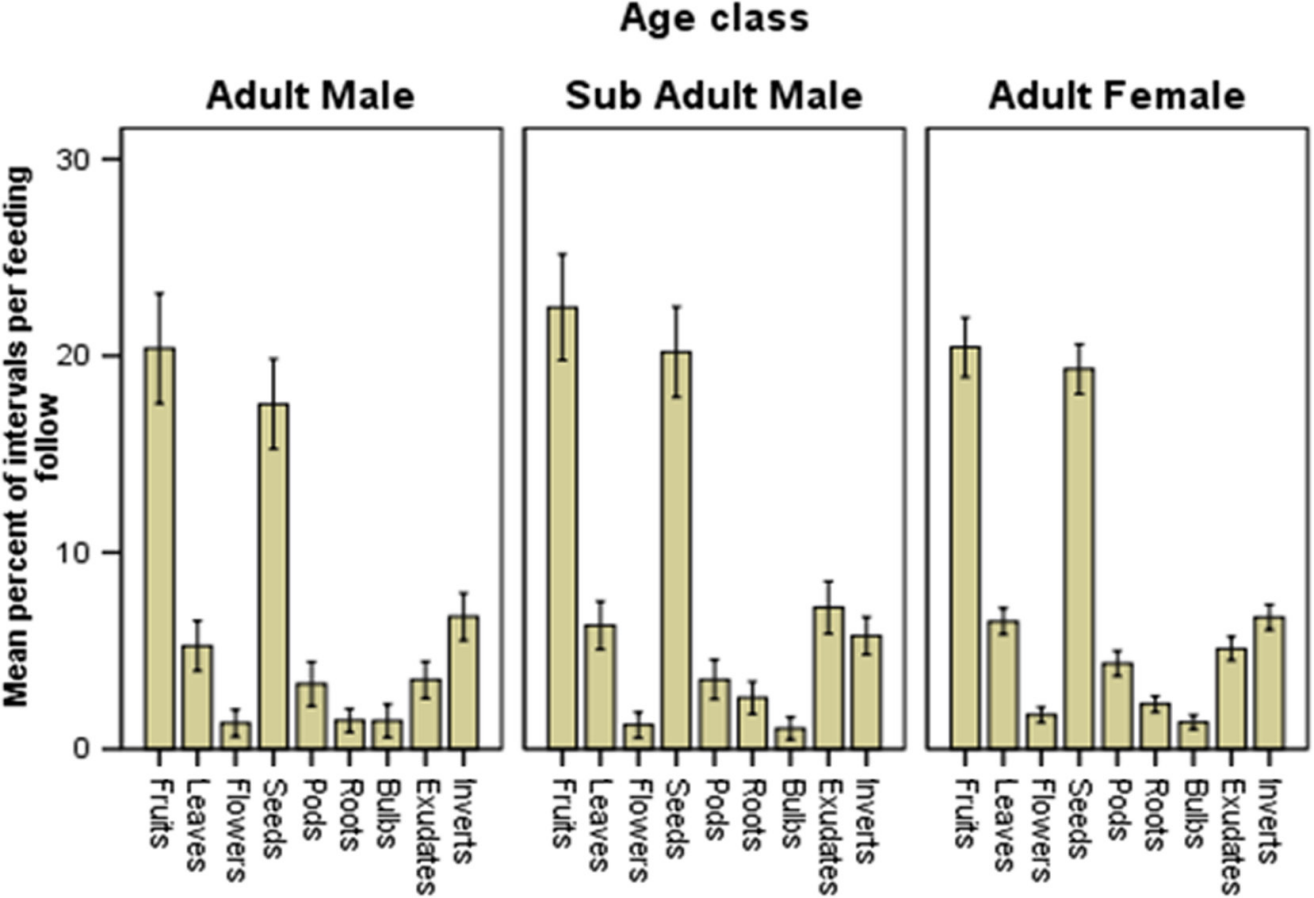
Mean percent of interval per feeding follow in the use of food parts per age and sex classes of yellow baboons: Adult Males [*N* = 557], Adult Females [*N* = 1862] and Subadult Males [*N* = 655]; Error bar = standard error of mean.

The diet of baboons was recorded to be composed of different plant parts i.e., plant leaves, seeds, fruits, and sap. Most frequently in a single meal, the diet was dominated by seeds and fruits while based on availability, invertebrates and sap (plant exudates) had significant contribution in the diet (Figure 7). Age and sex differences had no significant contribution in variation of diet composition except on the inclusion of plant exudates/sap. Adult males included more exudates in their diet than both adult females and subadult males (Table 4, test of model effects).

**TABLE 4:** Results of Generalized Estimating Equation (GEE) analysis on food part compositions analyzed as the consumption of food item in intervals per feeding follow by age‐sex classes of yellow baboons (SAM, Subadult males; AF, Adult Females and AM, Adult males).

Test of Model effects					Parameter Estimates					
Response variable	Wald Chi‐Square	df	*N*	*p*‐value	B‐coefficient			*p*‐value		
					AM vs AF	SAM vs AF	SAM vs AM	AM vs AF	SAM vs AF	SAM vs AM
Fruits	1.627	2	3074	.443	−0.004	0.120	0.124	.970	.215	.306
Leaves	2.289	2	3074	.319	−0.226	−0.034	0.193	.131	.783	.272
Flowers	2.079	2	3074	.354	−0.277	−0.358	−0.082	.340	.223	.829
Seeds	2.914	2	3074	.233	−0.118	0.054	0.173	.216	.409	.089
Pods	3.461	2	3074	.177	−0.282	−0.219	0.063	.180	.142	.792
Roots	5.312	2	3074	.070	−0.456	0.139	0.595	.036	.450	.028
Bulb	0.501	2	3074	.778	0.053	−0.264	−0.318	.890	.500	.540
Exudates	12.612	2	3074	.002	−0.388	0.365	0.753	.020	.023	.000
Inverts	1.695	2	3074	.429	0.003	−0.163	−0.166	.982	.208	.312

## 4 DISCUSSION

Foraging behavior of an animal can largely be influenced by the abundance and availability of food resources, which in turn can be affected by the environment in which the animal is living (Ménard et al., 2013). In this study, we investigated whether the TANZAM highway which traverses Mikumi National Park for over 50 km is an important source of food for yellow baboons. We set and analyzed data on three hypotheses to understand the costs and benefits attached to the use of highway during feeding and foraging by yellow baboons. First, we hypothesized that baboons used the highway for ease of movement through parts of their home range; second, we hypothesized that baboons used the highway because it improves visibility and reduces predation risk when moving within the home range; and third, we hypothesized that, high quality, easy to process domestic food items dropped on the highway, make it energetically profitable to travel and feed along the highway. These hypotheses were set based on the notion that yellow baboons in MINAPA spend more time on the highway waiting for spillover from agriculture produce and food leftovers thrown by passengers. Thus, the TANZAM highway serves as their major source of food.

### 4.1 Use of highway for ease of movement through parts of their home range

Findings on the frequency of visitation and time spent by baboons in different natural habitats and highway were used to test for this hypothesis. Natural habitats cover about 99% of total home range of 135 km^2^ of the study groups in MINAPA, thus the observed higher frequency of visitations to these habitats than to the highway, which covers <0.5% of the home range was expected. However, based on the frequency of visitation compared to proportion of home range area covered, baboons used wooded grassland, riverine and highway habitats more than expected, but used grassland and woodland less than was expected (Table 1). This is because, patterns of habitat use and movement of an animal has been designed to maximize nutrients capture over cost of time and energy (optimal foraging theory) (Bautista et al., 2001). For primates, the ecological influence on the pattern of their movement and habitat selection has been widely discussed (e.g., Barton et al., 1992; Chapman, 1988; Terborgh, 1983), and to larger extent the agreement is that yellow baboons' use of habitat and movement pattern are highly influenced by a number of factors such as adequate food and water supply, safety from predators, and suitable places for night resting (refugia) (Barton et al., 1992; Henzi et al., 2011). Two reasons can explain the unexpected observation of visitation and time spent in three habitats; wooded grassland, grassland, and woodland in relation to their availability. First, is the availability of food resource in the habitat; wooded grassland is the habitat mostly composed of woodland and grassland. Given the fact that yellow baboons are omnivorous, feeding on a wide variety of foods, a habitat with mixture of vegetation provide them with variety of food resources including grass seeds, leaves, roots, fruits, and exudates from trees. This explains the observed higher visitation (Table 1) and time spent on wooded grassland (Table 2) by yellow baboons in MINAPA despite that it covers only 14% of their home range. On other hand, although grassland and woodland cover a large part of yellow baboons' home range; they were likely to provide yellow baboons with limited food resources. For instance, woodlands were highly seasonal in food resource availability, and became more productive and suitable feeding habitat during the wet season (Alberts et al., 2005; Wessling et al., 2015). This has been also observed in feeding behavior of Chacma baboons (*Papio ursinus*) in woodland habitats in South Africa (Gaynor, 1994; Hill et al., 2003). Second, feeding in grassland is relatively safe for animals that are always taller than grasses as they can visualize the enemy from a distance; but for yellow baboons in Mikumi where grasses especially during wet season, are tall, feeding and spending time in grassland and even woodland become unsafe as hiding from predators like leopard (*Panthera pardus*) become difficult. Thus, yellow baboons tend to avoid frequent visitation to these habitats although they cover more than 70% of their home range.

The TANZAM highway traversing MINAPA passes near most of sleeping sites and water sources used by yellow baboons in Mikumi. The use of this highway by yellow baboons to and from their sleeping sites and feeding refugia explain the observed relatively high frequency of visitation to this habitat relative to their availability (cover less than 0.5% of the baboons' total home range) (Table 1). Moreover, observed higher visitation in the early morning (Figure 2) may be reflected by the fact that yellow baboons, on descending from sleeping tree they sit in small groups grooming, feed, and forage near sleeping sites for some time. Then, they move off while feeding and foraging around before resting during the day. Similar findings have been reported for Guinean baboons (*Papio papio*) (Anderson & McGrew, 1984; Fischer et al., 2017), Hamadryas baboons (*Papio hamadryas*) (Schreier & Swedell, 2008), and Chacma baboons (*Papio ursinus*) (Barton et al., 1992; Cowlishaw, 1996; Hill et al., 2003; Sick et al., 2014). Yellow baboons being on the highway most frequently in the early afternoon to mid‐afternoon (Figure 2), is likely to be their response to mid‐day high temperature. Although during hot time of the day yellow baboons generally never actively seek shade (Stelzner, 1988), but require access to water as their effective mechanism for brain cooling (Brain & Mitchell, 1999; Hill, 2006). In MINAPA, most of the permanent water sources are close to the highway thus yellow baboons spend some time along the highway when moving toward water sources. Moreover, the openness of the highway provided more visibility and reduced predation risks, hence moving toward water sources through highway was the safest avenue. This may also reflect the fact that adult females and subadult males, both with smaller body size were the most frequent visitors of the highway than larger bodied adult males.

### 4.2 The highway is used because it improved visibility and reduce predation risk when moving and feeding within the home range

In ecology of prey animals, habitat structure is important in determining the level of predation risk involved. The prey animal may alter its behavior especially activity budget and movement pattern in response to perceived predation risk “landscape of fear” (Coleman & Hill, 2014; Laundré et al., 2010). Perceived predation risk may have profound effect on activity budget especially foraging behavior, an animal may reduce time spent or avoid feeding on high‐risk areas and increase vigilance (Laundré et al., 2010). This may reflect the observed fact that yellow baboons spent more time than expected in wooded grassland and enough time on the highway, but spent little time than expected in woodland and grassland (Table 2). Moreover, baboons spent about 56% of their time in areas that cover less than 15% of their total home range (wooded grassland and highway) (Table 2), suggesting that the observed pattern of habitats use represented actual choice options by yellow baboons. On the other hand, woodlands in Mikumi are dominated by *Brachystegia*, *Combretum*, or *Terminalia* spp (Miombo woodland); and grassland dominated by *Themeda triandra*. Such vegetation increases the perceived predation risks of these habitats because short trees of Miombo woodlands and tall *Themeda triandra* grasses of grassland make it difficult for yellow baboons to detect predators. This, explains why yellow baboons used these habitats less than expected regardless of their relative contribution to their home range. Additionally, the time spent by yellow baboons in these habitats may reflect the importance of these habitats in relation to behavior performed. When using smaller recording intervals, one–zero results correlate well with continuous measures of frequency and duration, making it relatively easier to estimate the duration of behavior occurrence (Suen & Ary, 1984).

Although time spent feeding and foraging by yellow baboon is highly influenced by availability and distribution of food patches as well as troop size, the fact that baboons spent relatively similar proportion of time feeding and foraging in different habitats (Table 2) implies that, no matter the difference in vegetation, feeding occupies higher percent of the activity budget of yellow baboons. Such finding has been widely documented in studies related to feeding and foraging behavior of other baboon species (Cowlishaw, 1996; Hill & Dunbar, 2002; Norton et al., 1987; Post, 1982; Rhine et al., 1989; Whiten et al., 1991). The lower proportion of feeding in the riverine forests is influenced by the nature of its vegetation (mostly ditches and trees), which is mostly suitable for sleeping and drinking rather than foraging.

Moreover, in addition to feeding, when on the highway, baboons were also frequently engaged in socializing and resting particularly adult females and subadult males (Figures 3 and 4). This is because the openness of the highway contributes to perceived low predation risks. When animals are feeding in areas with lower perceived predation risks, they will increase their feeding time as well as resting time to allow digestion (landscape of fear theory) (Laundré et al., 2010). The highway is a non‐natural habitat, and yellow baboons were feeding on both natural and non‐natural foods, such as small‐sized cash crops produce, domestic fruits, and/or spill of human ready‐made foods. Feeding on such easy to process foods enabled yellow baboons to have ample time for resting and socializing. This is in consistence with the finding from Altmann and Muruthi (1988) on feeding behavior of yellow baboons in Amboseli National Park where they were reported to increase their time for resting and socializing when feeding from human leftovers.

Feeding and foraging behavior of yellow baboons is associated with other activities including vigilance (many eyes hypothesis). Yellow baboons were observed to have a higher frequency of vigilance when feeding in grassland and woodland, while having similar frequencies of vigilance in open woodland and on the highway (Figure 5). This observation could be explained in two ways; first, the high vigilanting behavior when feeding on grassland is for safety reasons. Grassland in Mikumi is composed of taller grasses, which hinder visibility of predators. Secondly, when feeding on the highway, baboons were also frequently socializing and resting (Figure 4) They were observed moving in fewer follows on the road (Figure. 4) while vigilance occurred with a similar frequency to vigilance in the adjacent and most visited habitat; wooded grassland. The similarity in feeding patterns (Table 2) and in vigilance (Figure 6) with frequently visited adjacent habitats (Table 2, Figure 6) suggests that the few visits recorded on the highway are not a direct consequence of using the highway as a safe vigilance platform. That movement occurred in a much lower number of follows on the road and the temporal use patterns (Figure 3) are consistent with movement out of sleeping trees in the early morning and movement to waterholes in the early afternoon. Moreover, in Mikumi solitary males who can invade the troop and most often found wandering along the highway, and this is likely to explain the observed higher vigilance behaviour of males than females and subadults when on the highway (Figure 7).

### 4.3 High quality easy to process domestic food items make it energetically profitable to travel and feed along the highway

Yellow baboons' diet was composed of variety of foods mainly from plants including, but not limited to fruits, seeds, flowers, leaves, pods, bulbs, roots, sap/ exudates, and animals mostly invertebrates (the whole animal or their products). These foods were mostly collected from natural habitats, but in some incidence from the highway as spillovers of cash crops (seeds and fruits), and/or anthropogenic waste food. Feeding of the highway food items by yellow baboons is because of seasonality in plant productivity and phenology. Baboons as omnivorous may consume variety of food whenever an opportunity arises (Norton et al., 1987). Regardless of the feeding habitats, whether natural or on the highway, fruits and seeds were the major component in baboons' diet; however, when feeding in natural habitats, leaves, sap/exudates and invertebrates were important inclusion in the diet. This is because nutritionally, fruits contain easy and readily available carbohydrates making them higher in energy value (Kamilar & Pokempner, 2008; Kunz & Linsenmair, 2010; Post, 1982). Therefore, fruits serve as the primary source of readily available energy for baboons regardless of sex and morphological size differences (Agetsuma, 2001; Kunz & Linsenmair, 2007). Difference in nutritional requirement driven by difference in body size and sex explain the observed differences in supplementary diet. Because of reproduction role of female and continuous growing of subadult males, they both require higher quality diet (Isbell et al., 2013). Thus, females tend to supplement their fruit diet with arthropods, which are higher in protein and fats compared with leaves (Rhine et al., 1989; Rothman et al., 2014). Subadult males tend to suppliant their fruit diet with plant exudates because it is rich in carbohydrates and some minerals (Garber, 1984; Porter et al., 2009), hence provide much needed energy for growth. Male baboons usually feed on lower, but more abundant food, thus will tend to supplement their fruit diet with leaves and seeds that are more abundant and provide them with easy and readily available protein (Rothman et al., 2014).

It was established in this study that, when using one–zero with small interval of feeding bouts within a feeding follow, the frequency of feeding per overall feeding follow reflects the dietary composition of food parts in yellow baboons' diet. Thus, the observed lower inclusion of anthropogenic waste food (exotic foods in Table 3) in the diet of baboons could be explained by high plant biodiversity in Mikumi National Park. There are about 2000 plant species in MINAPA of which yellow baboons consume more than 10% (Norton et al., 1987). In addition, it could also be explained by the fact that such foods are rare along the TANZAM highway because of routine cleaning of the highway by park authorities and strict regulation on human feeding of animals and park littering. These findings nullify our predication that the highway is used by baboon in Mikumi because it is energetically profitable to travel and feed along it because of high quality easy to process domestic food items that could be obtained from there.

## 5 CONCLUSIONS

This study set three hypotheses and a number of predictions to scrutinize the importance of TANZAM on the feeding and foraging behavior of yellow baboons. This was done following the notion that TANZAM highway serves as their major source of food because casual observations suggest that yellow baboons spend more time on the highway waiting for spillover of agriculture produce and anthropogenic waste food thrown by passengers. Findings of this study provide empirical evidence that yellow baboons do not directly depend on the highway for food, rather they consider highway as the normal part of their home range, and they use when moving and feeding within the home range. This implies that in protected areas with reduced human activities and strict enforcement of regulations no littering and no feeding of animals; highway traversing protected areas apart from their direct negative impact through road mortality, may have little influence on feeding and foraging behavior of animals.

## AUTHOR CONTRIBUTIONS

**Amani Salum Kitegile:** Conceptualization (lead); data curation (equal); formal analysis (supporting); funding acquisition (supporting); investigation (lead); methodology (equal); project administration (equal); resources (supporting); supervision (equal); validation (equal); writing – original draft (equal); writing – review and editing (equal). **Shombe N. Hassan:** Conceptualization (supporting); formal analysis (equal); methodology (equal); supervision (supporting); validation (equal); writing – original draft (equal); writing – review and editing (equal). **Guy William Norton:** Conceptualization (equal); funding acquisition (lead); methodology (equal); project administration (lead); resources (lead); supervision (lead).

## CONFLICT OF INTEREST

The authors declare(s) that there is no conflict of interest of any form either commercial or non‐commercial, financial or professional conflict.

### OPEN RESEARCH BADGES

This article has earned an Open Data badge for making publicly available the digitally‐shareable data necessary to reproduce the reported results. The data is available in reference number https://doi.org/10.6084/m9.figshare.19430417.

## Data Availability

The data that support the findings of this study are openly available in Figshare at reference number https://doi.org/10.6084/m9.figshare.19430417 and Major dataset of Influence of highway, Dryad, Dataset, https://doi.org/10.5061/dryad.00000006c.
